# Purpura Fulminans With Digital Gangrene in Severe Plasmodium falciparum Malaria: A Case Presentation

**DOI:** 10.7759/cureus.102245

**Published:** 2026-01-25

**Authors:** Inês Palmares, Catarina Morgado, Francisco Dá Mesquita Faustino, Joana Silva, Paulo Freitas

**Affiliations:** 1 Critical Care Medicine, Hospital Professor Doutor Fernando Fonseca, Amadora, PRT

**Keywords:** acral ischemia, critical care, digital gangrene, malaria, plasmodium falciparum, purpura fulminans, severe malaria

## Abstract

Purpura fulminans and peripheral gangrene are rare and severe complications of malaria, resulting from microvascular thrombosis driven by endothelial injury, cytokine-mediated coagulation and depletion of anticoagulants. Because there are no effective strategies to prevent progression to gangrene, the most important part of the management is initiating effective antimalarial therapy as soon as possible. In this report, the authors describe the case of a 66-year-old woman who returned from Angola without antimalarial chemoprophylaxis and presented with cerebral malaria due to Plasmodium falciparum infection. Despite early intravenous artesunate, hyperbaric oxygen therapy and supportive care, she developed purpuric lesions on the feet that evolved to gangrene, although systemic recovery was achieved.

## Introduction

Purpura fulminans is an acute, life-threatening thrombotic disorder characterized by extensive tissue thrombosis and hemorrhagic skin necrosis, often accompanied by disseminated intravascular coagulation (DIC) and complicated by peripheral gangrene [1]. It is often due to sepsis related to meningococcal and streptococcal infection, but has been occasionally reported as a complication of malaria [2,3]. 

Purpura fulminans and peripheral gangrene are rare but severe complications of malaria, in which there is distal ischemia in the absence of vascular occlusive disease. They are usually associated with severe forms of malaria and accompanied by multiple organ failure and DIC [4]. They result from a complex interplay of endothelial injury caused by parasitized erythrocytes' adherence to vascular endothelium, inflammatory cytokines triggering the coagulation cascade and consumption of anticoagulants like protein C [5-7]. These changes cause disseminated microvascular thrombosis, leading to characteristic skin necrosis and hemorrhage [5-7].

There have been some published reports of purpura fulminans and peripheral gangrene associated with malaria, especially in adults [2,3,8,9]. Here, we describe a case of purpura fulminans with digital gangrene in a 66-year-old woman with Plasmodium falciparum severe malaria.

## Case presentation

A 66-year-old woman with a history of hypertension presented to the Emergency Department (ED) with a one-week history of progressive drowsiness. She had returned from Angola three weeks prior and took no antimalarial chemoprophylaxis. On first evaluation on ED, she has a Glasgow Coma Scale of 8, which quickly dropped to 3, requiring orotracheal intubation for airway protection. Concomitantly with hypotension requiring vasopressor support in low dose (maximum dose of 0.3 mcg/kg/min for three days). The only positive finding on physical examination was icteric sclerae. A complementary study was performed, which revealed: hemolytic anemia (hemoglobin 8 g/dL), thrombocytopenia, renal impairment (serum creatinine 1.94 mg/dL, urea 280 mg/dL), direct hyperbilirubinemia (total 6.6 mg/dL, direct 4.0 mg/dL) and elevation of inflammatory markers along with an elevated lactate of 3.2 mmol/L (Table 1). Thin blood smear confirmed the presence of Plasmodium falciparum malaria with 9.7% of parasitized erythrocytes. As the major disturbance was neurologic impairment, a head computed tomography scan and a lumbar puncture were also performed, showing no significant abnormalities. Additionally, blood cultures and HIV, HBV and HCV serologies were also negative. The diagnosis of cerebral malaria was established, and she was started on intravenous (IV) artesunate and admitted to the ICU.

**Table 1:** Laboratory test results

	Emergency Department	3rd day of ICU stay	18th day of ICU stay	Normal reference range
Haemoglobin	8.0 g/dL	7.0 g/dL	9.2 g/dL	12.0 - 15.0 g/dL
White blood cell count	19 600/L	26 400/L	6 400/L	4 000 - 10 000/L
Platelet count	94 000/L	188 000/L	255 000/L	150 000 - 410 000/L
D-dimer	18 674 µg/L	-	-	< 500 µg/L
Fibrinogen	4.3 g/L	-	-	1.8 - 3.5 g/L
Creatinine	1.9 mg/dL	4.0 mg/dL	0.7 mg/dL	0.5 - 0.9 mg/dL
Urea	280 mg/dL	332 mg/dL	21 mg/dL	< 50.0 mg/dL
Total bilirubin	6.6 mg/dL	4.8 mg/dL	0.7 mg/dL	≤ 1.2 mg/dL
Direct bilirubin	4.0 mg/dL	3.3 mg/dL	-	≤ 0.2 mg/dL
C-reactive protein	13.8 mg/dL	16.9 mg/dL	1.0 mg/dL	< 0.5 mg/dL
Procalcitonin	33.1 ng/mL	37.7 ng/dL	0.1 ng/dL	< 0.1 ng/mL
Haptoglobin	< 10.0 mg/dL	< 10.0 mg/dL	-	30.0 - 200.0 mg/dL
Lactate	3.2 mmol/L	2.8 mmol/L	0.7 mmol/L	< 1.8 mmol/L

**Figure 1:**
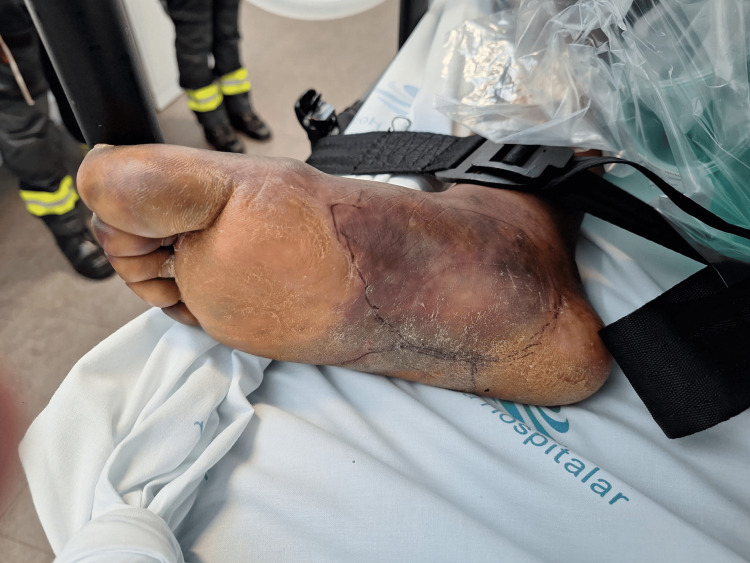
Purpuric lesions and digital ischemia of the right foot on the 4th day of ICU stay

**Figure 2:**
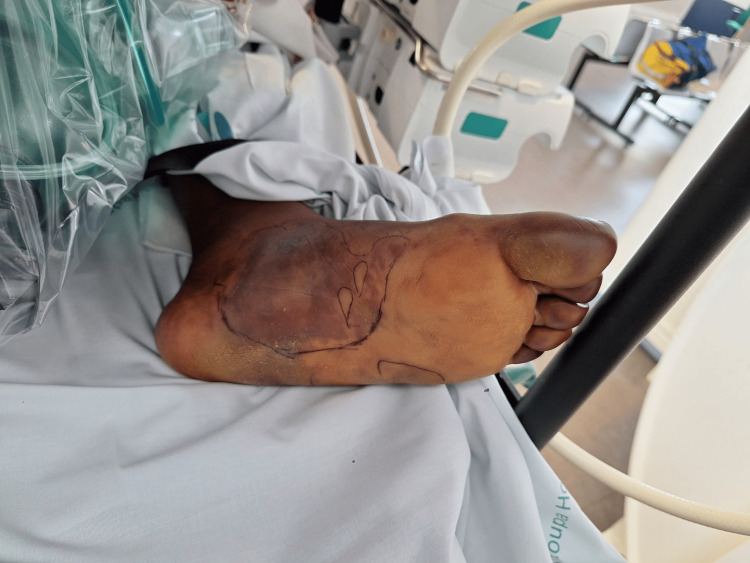
Purpuric lesions and digital ischemia of the left foot on the 4th day of ICU stay

On the fourth day of ICU, she developed purpuric lesions on both feet along with bilateral digital gangrene (Figures 1-2). Clotting times were normal, and there was no bleeding dyscrasia observed. An arterial doppler of the lower limbs revealed no abnormalities. She started hyperbaric oxygen therapy the next day and completed a total of six daily sessions without significant improvement in ischemia. General and Plastic Surgery teams were involved in a multidisciplinary discussion, and the patient was proposed for bilateral digital amputation, which she refused.

Completed 24 hours of IV artesunate therapy, parasitemia was <0.1%, but the oral route was still unavailable due to gastrointestinal intolerance with functional ileus and high residual gastric contents. She continued IV artesunate as IV doxycycline and quinine dihydrochloride were unavailable at the hospital, which was switched to clindamycin [10].

**Figure 3:**
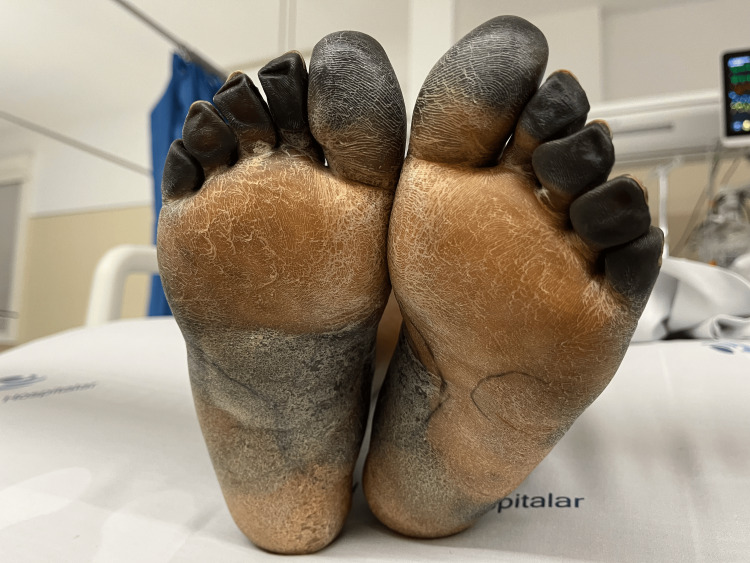
Digital dry gangrene on the 14th day of ICU stay

Neurologic recovery was attained, and she was successfully extubated on the twelfth day. Hematologic and renal dysfunction also improved. She received transfusion support with red blood cell concentrate, and acute kidney injury with preserved diuresis was managed with adequate fluid management. Despite overall recovery, digital gangrene of both feet remained (Figure 3), and eighteen days after admission, she was transferred to the surgical ward. After a month of hospitalization, she was discharged home against medical advice.

## Discussion

Malaria is a major cause of worldwide infection and mortality. The World Health Organization African Region continues to carry the heaviest burden of the disease and its mortality [11]. Of the four Plasmodium species responsible for malaria, Plasmodium falciparum infection is the most common and the most frequently associated with severe malaria [12].

The diagnosis of severe malaria is based upon the presence of one or more of the following clinical and laboratory criteria: cerebral malaria, metabolic acidosis, severe anemia, hypoglycemia, acute renal failure and/or acute pulmonary edema [13]. If left untreated, severe malaria is fatal in the majority of cases [13]. In our case, the severity of malaria was dictated by the impaired consciousness requiring invasive mechanical ventilation.

Purpura fulminans is often due to sepsis related to meningococcal and streptococcal infection, but has been occasionally reported as a complication of malaria [2,3]. It is associated with distal ischemia evolving to distal extremities gangrene requiring amputation surgery. The coagulopathy in malaria has been linked to multiple factors, including endothelial injury, activation of coagulation by inflammatory cytokines and a decrease in the concentration of anticoagulants [5-7]. The most important part of the management of coagulopathy secondary to malaria is the initiation of effective antimalarial therapy as soon as possible [4]. No specific treatment has been shown to consistently prevent progression or reverse gangrene [4]. Blood products should be administered to patients with DIC and spontaneous systemic bleeding, guided by coagulation tests [4]. Despite the prompt initiation of intravenous artesunate and hyperbaric oxygen therapy, our patient's digital ischemia gradually worsened and evolved to dry gangrene.

The management of malaria involves the eradication of the Plasmodium species and treatment of complications. For uncomplicated malaria infection, the treatment is an artemisinin-based combination therapy (ACT) [13]. Severe malaria should be treated with intravenous or intramuscular artesunate for at least 24 hours and until the oral route is available, which should be followed by a full dose of effective ACT orally [13].

## Conclusions

Purpura fulminans and peripheral gangrene are rare but devastating complications of severe malaria. Early identification and prompt antimalarial therapy are crucial, yet distal ischemia may remain irreversible despite appropriate treatment. These manifestations highlight the importance of early recognition of atypical presentations, timely initiation of antimalarial therapy and the need for preventive measures such as chemoprophylaxis in endemic areas. Greater awareness and reporting of such cases are essential to improve understanding and guide future management strategies.
